# Disturbed Pitch Perception during Antidepressant Therapy of a Combination of Lithium, Nortriptyline, and Oxazepam: A Rare Unexpected and Undesirable Side Effect for a Violinist

**DOI:** 10.1155/2022/4494284

**Published:** 2022-07-21

**Authors:** Siwert de Groot

**Affiliations:** University Groningen, Groningen, Netherlands

## Abstract

Disturbed pitch perception is a rare but well-known side effect of the antiepileptic drug carbamazepine and its derivates. A patient is presented who used three antidepressants because of depression. After recovering, the medication was continued, but as a violinist, the patient was told that his intonation was too low with the consequence that he was not allowed to participate in the orchestra where he had been for years. After phasing out the medications, his pitch perception returned to normal. This observation is unique as no other examples of this side effect are found in the literature in relation to antidepressants.

## 1. Introduction

Modification of pitch perception as a side effect of a drug that act on the nervous system is uncommon. Pitch is one of the primary auditory sensations, along with loudness and timbre. It is the perceptual correlate of the periodicity or repetition rate of an acoustic waveform [1]. Based mainly on Japanese publications, it has been shown that carbamazepine, usually used as an antiepileptic drug, may cause modification of this sensation. A recent literature review on 29 cases noted that 93% of the patients with this change had a Japanese origin [2]. This overrepresentation is possibly due to a specialized music training that children in Japan receive in order to develop a good pitch perception. Almost all of them were females, and more than half of them were 20 years of age or younger. It is not uncommon that a pitch has shifted down half a tone as was seen in more than half of the described patients. Sometimes, the tone shifts in an upward direction. Two-thirds of the described patients used carbamazepine for epilepsy but also for some other underlying diseases, such as bipolar disorder, emotional disorders, or trigeminal neuralgia. The interval between the administration of carbamazepine and the onset of the disturbance varied from several hours to 1–3 weeks [3]. It should be realized, however, that this side effect is very rare and is also described in isolated cases for the antiepileptic drugs oxcarbazepine and lacosamide [4, 5]. In this case report, we present a patient, a violinist, who was treated for depression with three different psychopharmaca. He was told that his intonation was too low, which prohibited him from being a member of an orchestra. It shows that modification of pitch perception may also be caused by drugs other than the described antiepileptics.

## 2. Case Report

A healthy 64-year-old man, working as a violinist with perfect pitch perception, is fairly acutely forced to stop his work because of depression. After a treatment of six months with the tricyclic antidepressant nortriptyline 50 mg two times per day (a blood level of 0.085 mg/ml) and oxazepam 20 mg three times per day, his depression did not respond. It was decided to add 1000 mg of lithium daily based on the blood level (a blood level of 0.73 mmol/L). After this addition, his depression disappeared within two months, but he had to continue the prescribed medication. The patient resumed his activities as a violinist and contributed to a sound recording with a male choir for a CD. During this recording process, accompanied by a church organ, he was pointed out by the sound director of the male choir that he was continuously playing too low and also with incorrect intonation. After several attempts to improve the purity, he was not able to do so and was refused to continue his performance.

The patient himself did not hear that he was playing too low and consequently impure. For a second opinion, the patient was referred to a university psychiatric center. He was advised to stop all the prescribed psychopharmaca according to a phase-out plan. After the medication was totally stopped, his pitch perception rapidly recovered in one month, which became clear in the next concert that he gave with a music group. The patient was able to continue his activities as a violinist. Hearing-related tests were unfortunately not done before, during, or after the medication.

## 3. Discussion

The disturbance in pitch perception in patients using antidepressants is not described in the literature. The reason might be that in the majority of the patients who do not make music, this disorder goes unnoticed because it is not a problem for them. However, the described patient got into trouble as a violinist during the music performance, as was also objectively apparent from the sound recording. Before using the antidepressant medication, he had never experienced this phenomenon. After the disappearance of depression, the suspicion rose that the deviant tone perception could be caused by the use of the prescribed psychopharmaca. Unfortunately, it will not become clear which of the three can be held responsible for the depicted side effect. More proof of this could be obtained if a blinded controlled experiment had been carried out on the patient as an N-1-trial, but this was omitted because of the risks connected with it [6]. The detection of the disturbed pitch perception cannot be seen apart from the instrument used by the musician. For string players, intonation (producing the right pitch) is one of the most difficult parts of playing the violin. Unlike instruments with keys and fixed pureness of tones, if the keys are touched, the tones of a violin are not fixed. There is a continuum of note pitches from which to choose along the length of a string. The accuracy of the intonation depends on the ability to discriminate small differences and to press the string in the right place on the fingerboard [7]. This practically means that playing as a violinist together with other instruments or with an orchestra is impossible through deviant sound perception and therefore incorrect intonation. The accompanying organ tones were heard lower by the patient with the consequence that his adjusted intonation was interpreted by others as too low; however, he tried to play purely. It is not clear what the underlying pathophysiological mechanism of the described side effect is. On the basis of available data, it is assumed that the anticonvulsant carbamazepine has a suppressive influence on the auditory pathways, peripherally in the organ of corti as well as centrally at the brain stem level [8]. In some cases, a disturbed electro-encephalogram was found [5, 9]. But the auditory pathway is complex, and from studies of pitch, the picture emerges that the normal perception of pitch and simple pitch sequences involves networks that include the auditory cortices and adjacent areas in the superior-temporal lobe [10]. Possibly, the Hesch gyrus, located adjacent to the anterolateral border of the primary auditory cortex along the superior-temporal gyrus, can be designated as a so-called pitch center because it is consistently identified as activated in pitch modulation [11].

Of the three antidepressants given to the described patient, lithium is the most neurotoxic. The induced neurotoxicity may occur during the initial few days of treatment as well as after years of maintenance therapy. It is still not unthinkable that the combination of the three drugs still played a role in this clinical picture [12]. Information at the Netherlands Pharmacovigilance Centre Lareb learned that the side effect of the pitch perception disturbance has not been reported so far. A search on VigiBase shows that the used antidepressants may give sporadically ear and labyrinth disorders but more specified disturbed pitch perception has not been mentioned [13]. This specific side effect in relation to the antidepressants lithium, nortriptyline, and oxazepam is till now not mentioned in the literature. An advanced search on PubMed did not give a link to the side effect of disturbed pitch perception in relation to this medication. The only link to pitch loss found is related to the antiepileptic drug carbamazepine.

Hearing loss caused by a combination of the three medicines is not mentioned in the literature either. It is known, however, that people with hearing loss, and especially those with cochlear implants, often suffer from a deficit in pitch perception abilities [1]. Other hearing side effects of the described antidepressant here are tinnitus and hyperacusis, although the effect on auditory perception could not be found in an experiment after the intake of oxazepam. Compared with a placebo group, benzodiazepines do not appear to exert a significant influence on selective attention or loudness perception, at least in the short term [14].

## 4. Conclusion

The problem of a disturbed pitch perception that the patient experienced as a violinist raised the suspicion that one or more of the antidepressants (nortriptyline, lithium, and oxazepam) could be responsible for it. This side effect has not been described in the literature until now. It remains, however, uncertain which of the three antidepressants may be mainly held responsible for the disturbance of intonation. Because of its neurotoxicity, lithium is the most suspicious. The message is that doctors as well as musicians should be aware of this side effect, particularly because, unlike carbamazepine, the use of antidepressants is not uncommon today.
